# Overexpression of long noncoding RNA LINC00638 inhibits inflammation and oxidative stress in rheumatoid arthritis fibroblast‐like synoviocytes by regulating the Nrf2/HO‐1 pathway

**DOI:** 10.1002/iid3.663

**Published:** 2022-06-20

**Authors:** Yanqiu Sun, Jian Liu, Jianting Wen, Dan Huang, Qin Zhou, Xianheng Zhang, Xiang Ding, Xiaolu Chen

**Affiliations:** ^1^ Graduate School Anhui University of Traditional Chinese Medicine Hefei Anhui China; ^2^ Department of Rheumatology The First Affiliated Hospital of Anhui University of Traditional Chinese Medicine Hefei Anhui China; ^3^ Institute of Rheumatology Anhui University of Traditional Chinese Medicine Hefei Anhui China

**Keywords:** inflammation, LINC00638, oxidative stress, reactive oxygen species, rheumatoid arthritis

## Abstract

**Background:**

Abnormal expression of long noncoding RNAs (lncRNAs) is involved in several autoimmune diseases including rheumatoid arthritis (RA). In this study, we intended to explore the expression of lncRNA LINC00638 in RA and its potential mechanism of action related to inflammation and oxidative stress.

**Methods:**

The level of LINC00638 in the peripheral blood mononuclear cells (PBMCs) obtained from 45 RA patients and 30 normal controls was analyzed and its correlation with clinical indicators was investigated. In vitro, we used tumor necrosis factor‐α to stimulate fibroblast‐like synoviocytes (FLS) of RA patients for cell based experiments. Subsequently, the overexpressed plasmid and small interfering RNA of LINC00638 were designed. Furthermore, we further analyzed the potential effects of LINC00638 on the proliferation and migration of RA‐FLS and the nuclear factor erythrocyte derived 2 related factor 2 (Nrf2)/heme oxygenase 1 (HO‐1) pathway.

**Results:**

LINC00638 expression was found to be significantly decreased in PBMCs of RA patients, and it was negatively correlated with erythrocyte sedimentation rate, interleukin (IL)‐17, reactive oxygen species (ROS), and disease activity scores for 28 joints (DAS28). Overexpression of LINC00638 activated the Nrf2/HO‐1 pathway, markedly decreased the expressions of IL‐6, IL‐17, IL‐23, ROS, as well as malondialdehyde, increased the total antioxidant capacity, and attenuated the proliferation and migration of RA‐FLS, while silencing of LINC00638 reversed these manifestations.

**Conclusions:**

LINC00638 was found to be expressed at low levels in RA patients and was associated with immune inflammation, oxidative stress, and disease activity. Overexpression of LINC00638 can reduce the proliferation as well as migration of RA‐FLS, and activate the Nrf2/HO‐1 pathway to inhibit the inflammation and oxidative stress.

## Graphical abstract

LINC00638 is down‐expressed in fibroblast‐like synoviocytes from patients with rheumatoid arthritis. Overexpression of LINC00638 can inhibit cell proliferation and migration, on the contrary, knockdown of LINC00638 can promote cell proliferation and migration.

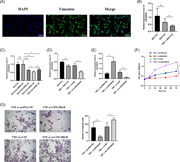

## 1 INTRODUCTION

Rheumatoid arthritis (RA) is a recurrent, refractory inflammatory disease that can cause significant joint destruction.^1^, ^2^ The causes of RA are unknown and may be related to the various factors such as heredity, infection, and environment.^3^ The basic pathological manifestations are that of synovial hyperplasia, synovial inflammation, and pannus formation.^4^ Fibroblast‐like synoviocytes (FLS) are the main effector cells involved in the process of joint destruction. Excessive proliferation and migration of FLS can lead to synovial inflammation and bone destruction by secreting a variety of inflammatory mediators.^5^, ^6^

A number of studies have confirmed that immune inflammation and oxidative stress can display cross talk with each other and participate in pathogenesis of RA.^7^, ^8^ For instance, excessive accumulation of reactive oxygen species (ROS) can lead to oxidative stress.^9^ The various inflammatory factors can promote the synovial joint proliferation as well as cartilage destruction and produce harmful free radicals. Oxidative stress can further promote joint damage by acting as a potential mediator of inflammation.^10^, ^11^ Nuclear factor‐erythroid 2‐related factor 2(Nrf2)/heme oxygenase 1 (HO‐1) signaling pathway can exhibit significant antioxidant and anti‐inflammatory effects that have been found to be closely linked to RA.^12^ Therefore, we focused on analyzing the upstream regulatory factors of this pathway in RA models.

Long noncoding RNA (lncRNA) can play a variety of role in various autoimmune diseases including RA.^13^, ^14^ Our group has previously demonstrated that expression of LINC00638 was correlated with immunoinflammatory index of RA patients by RNA sequencing,^15^ but there was a lack of clinical validation with limited sample size and mechanistic evidences about how this lncRNA can exert specific regulatory effects. In this study, we have examined the level of LINC00638 in peripheral blood mononuclear cells (PBMCs) in RA patients and normal control (NC). Furthermore, we have analyzed the correlation between LINC00638 and the clinical laboratory indicators of RA patients. In addition, we have investigated the potential effects of LINC00638 on the proliferation as well as migration of RA‐FLS and on the Nrf2/HO‐1 signaling pathway.

## 2 MATERIALS AND METHODS

### 2.1 Subjects

A total of 45 RA patients who meet the diagnostic criteria of RA classification were enrolled in this study.^16^ The patients were inpatients from the First Affiliated Hospital of Anhui University of Traditional Chinese Medicine. Patients with tumors, Sjogren's syndrome, or other autoimmune diseases were excluded. In addition, 30 subjects who were healthy and matched in gender and age were regarded as NC group. All the subjects signed an informed consent according to the Declaration of Helsinki. This study was approved by the Ethics Committee of the First Affiliated Hospital of Anhui University of Chinese Medicine (2019AH‐12). This study complied with STROBE guidelines. A total of 45 patients were hospitalized for RA for the first time, and none of them had used disease‐modifying antirheumatic drugs or glucocorticoids within 12 weeks before admission. Six patients had used nonsteroidal anti‐inflammatory drugs within 1 week before admission. In addition, none of the patients had taken any medication 3 days before the blood test.

### 2.2 Clinical data of the subjects

The clinical data of all subjects werr obtained through the hospital's medical management system. The general information included gender, age, course of the disease, number of joint swelling, and tenderness. The laboratory parameters that were measured included erythrocyte C‐reactive protein (CRP), rheumatoid factor (RF), erythrocyte sedimentation rate (ESR), anti‐cyclic citrullinate peptide antibody (anti‐CCP), and disease activity scores for 28 joints (DAS28).

### 2.3 Culture and identification of FLS

Synovial tissue samples were obtained from RA patients who had undergone joint replacement surgery. Briefly, the samples were cut into small fragments and cultured in Dulbecco's modified Eagle medium (DMEM) medium containing penicillin (100 U/ml) and streptomycin (0.1 mg/ml). The immortalized cell line of RA‐FLS was obtained by using lentivirus vector‐mediated SV40+ T antigen transfection.^17^ The second generation of the cells was used for identification, and the level of Vimentin protein was identified by immunofluorescence (IF) cytochemistry. RA‐FLS were thereafter fixed by paraformaldehyde and incubated with Triton X‐100 for 0.5 h. The primary anti‐vimentin (Bs‐8533R; Bioss) was then added and incubated at 4°C for 8 h. The secondary antibody (1:400, goat anti‐rabbit) was then added to all the sections and incubated for 1 h. After DAPI staining, the tablets were sealed and observed under a fluorescence microscope (BA410E; Motic). Based on this preliminary study, 3–5 generations of cells were selected for the subsequent experiments. FLS for NC (NC‐FLS, HUM‐iCell‐s010; iCell Bioscience Inc.) were cultured in DMEM medium containing 10% FBS (sh30256.01; Hyclone).

### 2.4 Cell transfection

Three different synthetic small interfering RNA (siRNAs) and negative control siRNA (si‐NC) of LINC00638 (LINC00638 #1, #2, #3) were constructed by researchers from Shanghai GenePharma Co., Ltd. Thereafter, we inserted the human lncRNA LINC00638 sequence into pcDNA3.1 plasmid and named it as pcDNA‐LINC00638. A plasmid carrying an untargeted control sequence served as a negative control for pcDNA‐LINC00638 and was named pcDNA‐NC. After RA‐FLS were cultured to adherence, they were stimulated with tumor necrosis factor‐α (TNF‐α) (20 ng/ml) for 24 h, and cells were transfected using the lipofectamine2000 (Invitrogen) transfection reagent. The experimental set up consisted of four different groups, including: TNF‐α + pcDNA‐NC, TNF‐α + pcDNA‐LINC00638, TNF‐α + si‐NC, and TNF‐α + si‐LINC00638. The cells were collected after 48 h of transfection for each of the above groups for the subsequent experiments.

### 2.5 Quantitative real‐time polymerase chain reaction (qRT‐PCR)

RNA was harvested using TRIzol agent and then reverse transcription kit was used to generate complementary DNA. NovostartR SYBR qPCR SuperMix Plus (E096‐01B, novoprotein) was employed for real‐time PCR. The sequence of the primers of LINC00638 used were as follows: 5′‐CCATAGCCGATTAGCTGTCA‐3′ (forward) and 5′‐AATGCCGAACTGGAGGTG‐3′ (reverse). β‐Actin was used as an endogenous control. The primers of LINC00638 were synthesized by Shanghai Sangon Biotech Technology Service Co., Ltd. in China.

### 2.6 Enzyme‐linked immunosorbent assay (ELISA)

The levels of the various cytokines such as IL‐6 (JYM0140Hu), IL‐17 (JYM0082Hu), IL‐23 (JYM0077Hu), and HO‐1 (JYM1097Hu) were measured using ELISA kits (Wuhan Genemei Biotechnology Co., Ltd.) in the serum or cell culture supernatant, according to manufacturer's instructions.

### 2.7 Measurement of ROS and lipid peroxide

Following the manufacturer's instructions, a commercial kit (475015; BestBio) was used to measure serum ROS by the chemiluminescence method. Biochemical kits (A003‐1, NanJing JianCheng Bioengineering Institute) were used to detect malondialdehyde (MDA) and total antioxidant capacity (TAOC, A015‐2‐1, NanJing JianCheng Bioengineering Institute) in the serum or cell supernatants.

### 2.8 Cell counting kit‐8 (CCK‐8) assay

The cell proliferation ability of RA‐FLS was measured by using CCK8 kit (BA00208; BIOSS). Initially, 3 × 10^4^ RA‐FLSs were inoculated in each well and incubated to an extent of 70–90% fusion. The RA‐FLS in the logarithmic growth phase were thereafter grouped and transfected. Three different repeat wells were set for each group and incubated for 0, 12, 24, 48, and 72 h, respectively. At each of the above experimental time points, 10 μl CCK‐8 solution was added into each well and incubated for 1.5 h at 37℃. Finally, the absorbance value of each well was measured at 450 nm.

### 2.9 Transwell assay

The migration ability of RA‐FLS was analyzed in a Transwell chamber. RA‐FLS were first suspended in the serum‐free medium, thereafter the cell concentration was adjusted to 2 × 10^6^/ml, and then 100 µl of cell suspension was inoculated in an upper chamber. In addition, the lower chamber was filled with 800 µl of DMEM medium. In this experiment, the cells in the upper chamber were removed after being placed in a 37℃ incubator for 48 h. The migrated RA‐FLS were immobilized and stained with crystal violet (G1062; Solarbio) for 20 min. Finally, three fields were randomly observed to calculate the average migration number of RA‐FLS.

### 2.10 Flow cytometry

According to the instructions provided in ROS kit (S0033S; Beyotime), the cells of each group were collected, and then suspended in the serum‐free culture medium. We thereafter added DCFH‐DA at a concentration of 10 μmol/L and the concentration of RA‐FLS used was 1 × 10^6^/ml. Finally, the ROS expression in RA‐FLS was determined by flow cytometer (MACSQuant Analyzer; Miltenyi Biotec).

### 2.11 Immunofluorescence

RA‐FLS was fixed in the cold acetone for 10 min and then in 0.1% Triton X‐100 permeable membrane for 0.5 h. Thereafter, the goat serum was added to the block for 0.5 h. After removing the goat serum, anti‐Nrf2 (1:400, SC‐365949; Santa Cruz), and anti‐HO‐1 (1:400, SC‐390991; Santa Cruz) antibodies were added and incubated at 37℃ for 1 h. All the sections were stained with corresponding secondary antibodies (1:400) and incubated for 20 min followed by DAPI (C1005; Beyotime) staining for 5 min. Finally, the fluorescence images of cells were captured under a fluorescence microscope (BA410E; Motic).

### 2.12 Western blot analysis

RIPA (P0013B; Beyotime) was used to extract the protein of RA‐FLS for protein quantification. Thereafter, the protein was separated by sodium dodecyl sulfate–polyacrylamide gel electrophoresis and transferred to the immobilized polyvinylidene difluoride membrane. Thereafter, 5% skim milk powder was used for blocking for 1 h. After washing, the primary anti‐Nrf2 (1:1000; Santa Cruz) and anti‐HO‐1 antibodies (1:1000; Santa Cruz) were added and incubated at 37°C for 1h. The membranes were then incubated with an HRP‐labeled secondary antibody (1:2000). After washing, ECL was used for development and the films were exposed, fixed, and the pictures were captured in the darkroom. LaminB1 or glyceraldehyde‐3‐phosphate dehydrogenase were used as internal controls and the absorbance value of the target protein was calculated.

### 2.13 Statistical analysis

In this study, SPSS 22.0 (SPSS Inc.) was used to analyze the data and the statistical results were expressed as mean ± standard deviation or P50 (P25, P75). Graphpad Prism 8.0 was used to draw the graphs. The Chi‐square test was used to compare the categorical variables. The significance of differences between the groups was determined by the Student *t* test. When *p* < .05, the difference was considered as statistically significant.

## 3 RESULTS

### 3.1 Demographic characteristics and clinical data of the subjects

In this study, 11 males (24.44%) and 34 females (75.56%) were included in RA group, with an average age of (55.98 ± 10.59) years, a median course of disease of 6.00 (2.00, 11.00) years, and an average DAS28 (5.71 ± 0.86) points. In the NC group, there were 7 males (23.33%) and 23 females (76.67%), with an average age of (56.23 ± 6.63) years. The two groups were matched in both age and gender (*p* > .05). The positivity of RF, CRP, ESR, and anti‐CCP in RA patients was found to be more than 75% (Table 1).

**Table 1:** General clinical characteristics of the subjects

Indicators	RA (*N* = 45)	NC (*N* = 30)	*t*/*χ* ^2^	*p* value
Male, *n* (%)	11 (24.44%)	7 (23.33%)	0.012	.912
Female, *n* (%)	34 (75.56%)	23 (76.67%)		
Age (years)	55.98 ± 10.59	56.23 ± 6.63	−0.128	.898
Duration of disease (years)	6.00 (2.00, 11.00)	–	–	–
Joint tenderness, *n*	9.47 ± 3.71	–	–	–
joint swelling, *n*	5.00 (2.50, 7.50)	–	–	–
DAS28	5.71 ± 0.86	–	–	–
RF positive, *n* (%)	44 (97.78%)	–	–	–
CRP positive, *n* (%)	35 (77.78%)	–	–	–
ESR positive, *n* (%)	44 (97.78%)	–	–	–
anti‐CCP positive, *n* (%)	40 (88.89%)	–	–	–
RF (U/ml)	113.00 (47.70, 233.10)	–	–	–
CRP (mg/L)	15.00 (5.36, 39.84)	–	–	–
ESR (mm/h)	34.00 (23.00, 54.50)	–	–	–
anti‐CCP (U/ml)	103.00 (36.75, 323.00)	–	–	–

Abbreviations: anti‐CCP, anti‐cyclic citrullinate peptide antibody; CRP, C‐reactive protein; DAS28, disease activity score of 28 joints; ESR, erythrocyte sedimentation rate; RF, rheumatoid factor.

### 3.2 Levels of LINC00638, serum inflammatory cytokines, and oxidative stress markers in RA patients

**Figure 1:**
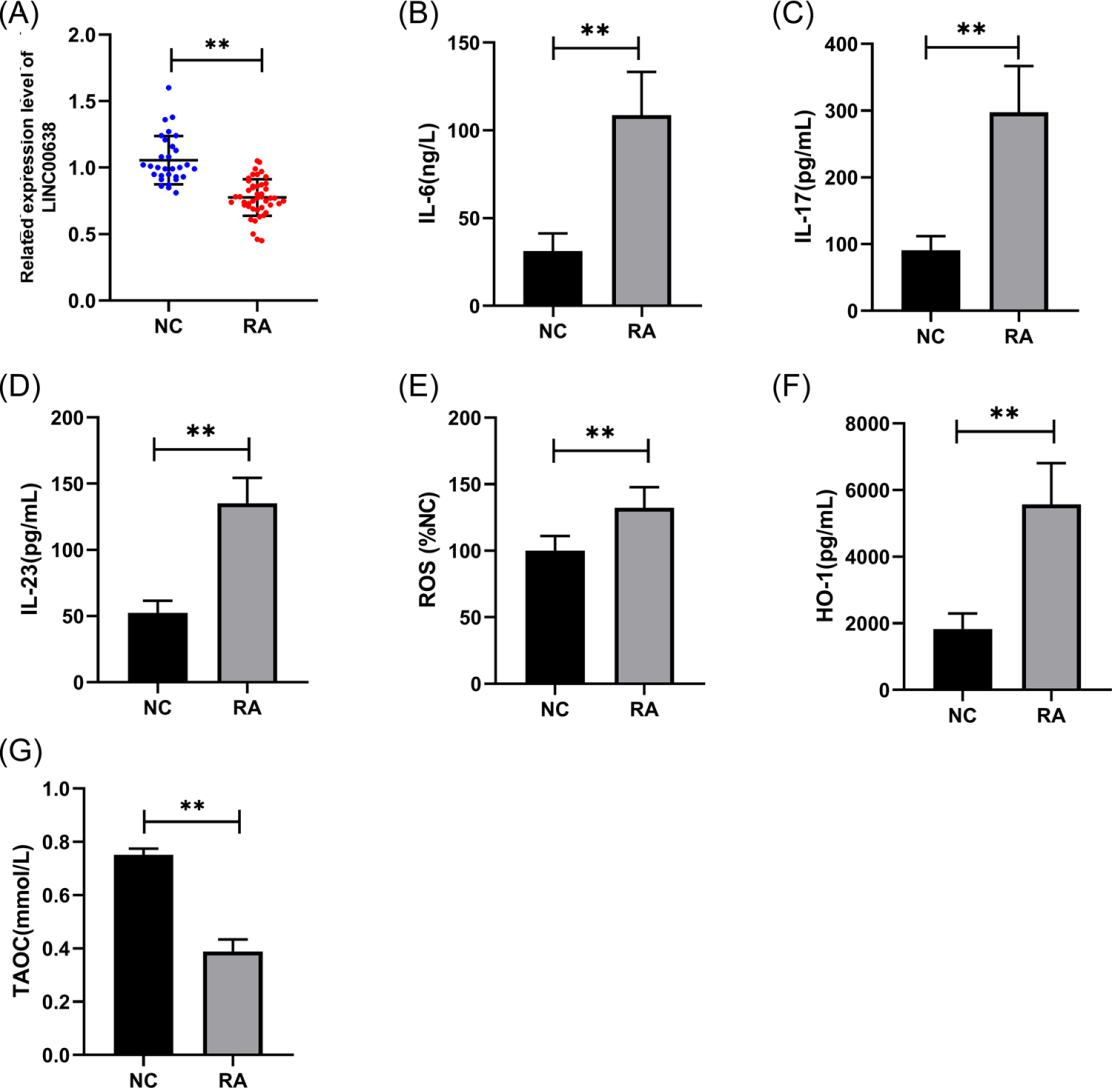
Levels of LINC00638 in PBMC and serum inflammatory cytokines and oxidative stress markers in RA patients. (A) The expression of LINC00638 in PBMCs of subjects by qRT‐PCR. (B–D) ELISA method was used to detect increased levels of IL‐6, IL‐17, and IL‐23 in the serum of RA patients. (E) Chemiluminescence assay detected increased the expression of ROS in the serum of RA patients. (F) ELISA method detected increased levels of HO‐1 in the serum of RA patients. (G) Biochemical assay was employed to measure decrease of TAOC in the serum of RA patients. IL‐6, interleukin‐6; IL‐17, interleukin‐17; IL‐23, interleukin‐23. **p < .01. ELISA, enzyme‐linked immunosorbent assay; HO‐1, heme oxygenase 1; IL, interleukin; PBMC, peripheral blood mononuclear cell; qRT‐PCR, quantitative real‐time polymerase chain reaction; RA, rheumatoid arthritis; ROS, reactive oxygen species; TAOC, total antioxidant capacity

To investigate LINC00638 level, qRT‐PCR was performed in PBMCs of subjects. The findings indicated that the expression of LINC00638 was significantly reduced in RA patients (Figure 1A). Subsequently, to further assess the levels of inflammation and oxidative stress markers in RA patients, we examined the expression of inflammatory cytokines, ROS, and antioxidant enzymes. The results showed that the levels of interleukin (IL)‐6, IL‐17, IL‐23, ROS, and HO‐1 were significantly increased whereas that of TAOC was decreased in the serum of RA patients compared with the NC group (Figure 1B–G). These observations suggested the presence of high levels of inflammatory response and oxides in RA patients, but they appeared to display insufficient antioxidant capacity.

### 3.3 Levels of LINC00638 in PBMC were associated with inflammation and oxidative stress in RA patients

The correlations were analyzed using the Spearman correlation between LINC00638 and clinical indicators, cytokines and oxidative stress indicators. It showed that the level of LINC00638 was negatively correlated with DAS28 (*r* = −.344, *p* = .021), ESR (*r* = −.480, *p* = .001), IL‐17 (*r* = −.321, *p* = .031) and ROS (*r* = −.547, *p* < .001) in RA patients. There was no significant correlation observed between the level of LINC00638 and other indicators (Table 2).

**Table 2:** Correlation between LINC00638 and laboratory indicators in RA patients

Indicators	*r*	*p* value
Age (years)	−.091	.554
Duration of disease (years)	−.054	.726
DAS28	−.344	.021
RF (U/ml)	.091	.551
CRP (mg/L)	.155	.311
ESR (mm/h)	−.480	.001
anti‐CCP (U/ml)	.056	.717
IL‐6 (ng/L)	−.266	.078
IL‐17 (pg/ml)	−.321	.031
IL‐23 (pg/ml)	.265	.078
ROS (Ex/Em = 488/520 nm)	−.547	<.001
HO‐1 (pg/ml)	−.133	.384
TAOC (mmol/L)	−.030	.845

Abbreviations: anti‐CCP, anti‐cyclic citrullinate peptide antibody; CRP, C‐reactive protein; DAS28, disease activity score of 28 joints; ESR, erythrocyte sedimentation rate; HO‐1, heme oxygenase 1; IL‐6, interleukin‐6; IL‐17, interleukin‐17; IL‐23, interleukin‐23; RF, rheumatoid factor; ROS, reactive oxygen species; TAOC, total antioxidant capacity.

### 3.4 LINC00638 can regulate the proliferation and migration of TNF‐α‐stimulated RA‐FLS

**Figure 2:**
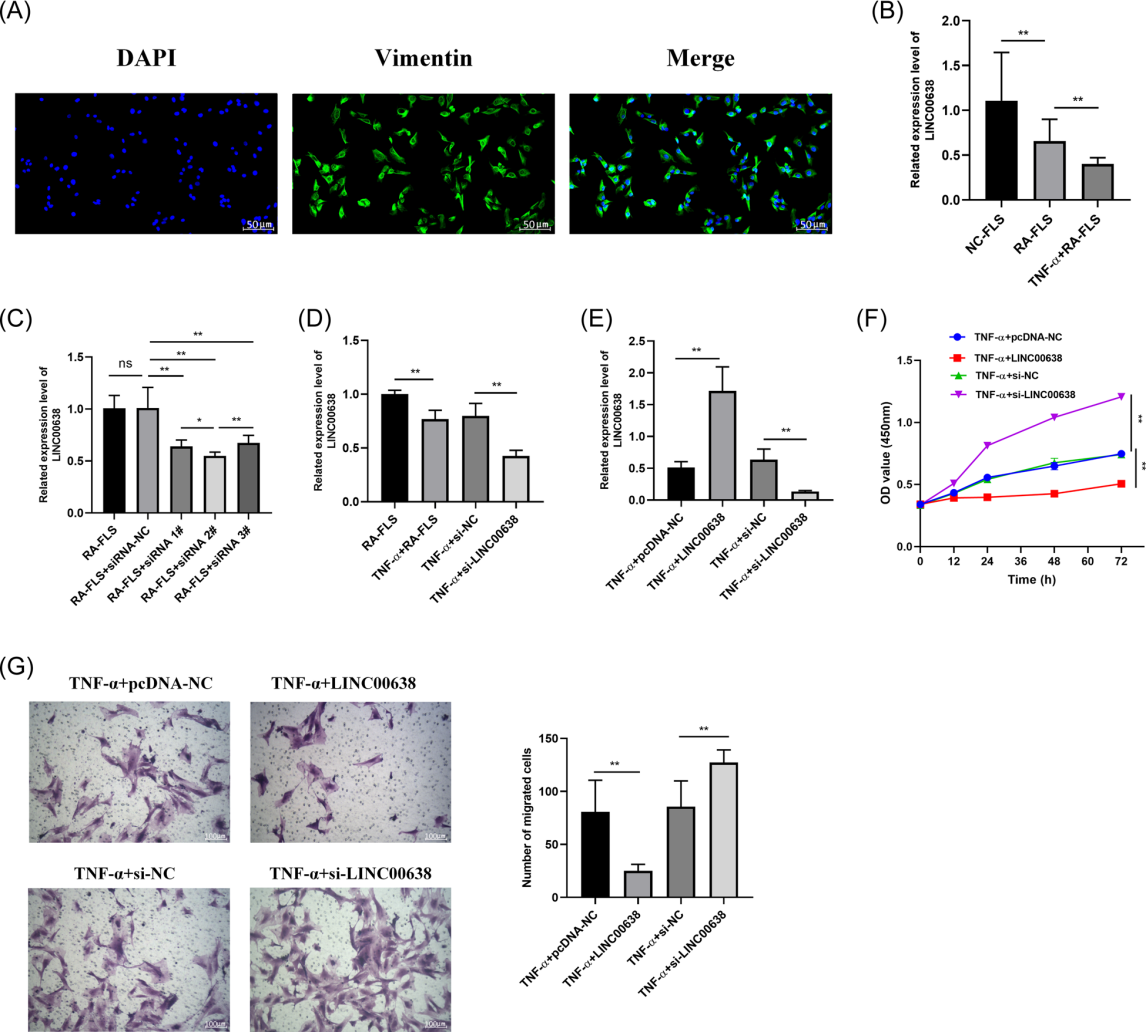
LINC00638 regulates the proliferation and migration of TNF‐α‐stimulated RA‐FLS. (A) The immunofluorescent staining of RA‐FLS with vimentin antibody (green), and DAPI stained blue. fluorescence represents the nucleus (scale bar, 50 μm). (B) Expression of LINC00638 in NC‐FLS, RA‐FLS, and TNF‐α‐stimulated RA‐FLS. (C) The expression level of LINC00638 in RA‐FLS transfected with si‐LINC00638. (D) Expression of LINC00638 in RA‐FLS stimulated by TNF‐α transfected with si‐LINC00638. (E) Expression of LINC00638 in TNF‐α‐stimulated RA‐FLS transfected with overexpression plasmid or siRNA. (F) The potential effects of LINC00638 on the proliferation of TNF‐α ‐stimulated RA‐FLS as measured by CCK8 assay. (G) Effect of LINC00638 on the migration of RA‐FLS stimulated by TNF‐α as analyzed by Transwell assay (scale bar, 100 μm). ns: no significance, *p < .05, and **p < .01. ELISA, enzyme‐linked immunosorbent assay; HO‐1, heme oxygenase 1; IL, interleukin; PBMC, peripheral blood mononuclear cell; qRT‐PCR, quantitative real‐time polymerase chain reaction; RA, rheumatoid arthritis; ROS, reactive oxygen species; TAOC, total antioxidant capacity; TNF, tumor necrosis factor

IF assay was used to detect the expression of vimentin (an important marker of FLS) on the second‐generation cells. Under fluorescence microscopy, the cytoplasm was tagged with vimentin is green, and the nucleus was tagged with DAPI is blue (Figure 2A), which indicated that the cultured cells were RA‐FLS.

The expression of LINC00638 in RA‐FLS was noted to be lower than that in normal synovial fibroblasts (NC‐FLS), which was consistent with the data observed in PBMCs (Figure 2B). Subsequently, after TNF‐α (20 ng/ml) stimulated for 24 h, the expression of LINC00638 in RA‐FLS was significantly reduced (Figure 2B). In addition, the expression of LINC00638 was markedly decreased in the RA‐FLS transfected with si‐RNA (1#, 2#, and 3#), but we selected si‐RNA 2# with the highest inhibition efficiency for the follow‐up experiments (Figure 2C). Moreover, LINC00638 level was lower in RA‐FLS transfected with si‐RNA2# after TNF‐α stimulation (Figure 2D). Therefore, we used TNF‐α to stimulate RA‐FLS as a cell model for the subsequent experimental studies.

By detecting the expression of LINC00638, we successfully transfected LINC00638 overexpression plasmid or siRNA in RA‐FLS (Figure 2E). To observe the potential effect of LINC00638 on the biological behavior of TNF‐α‐stimulated RA‐FLS, the proliferation of RA‐FLS was detected by CCK8 assay and the cell migration was detected by Transwell assay. The results showed that compared with RA‐FLS induced by TNF‐α, the cells transfected with pcDNA‐LINC00638 exhibited significantly lower proliferation and cell migration capabilities (Figure 2F,G). In contrast, RA‐FLS transfected with si‐LINC00638 showed relatively higher proliferation and migration ability (Figure 2F,G).

### 3.5 LINC00638 can regulate the levels of inflammatory cytokines and oxidative stress in TNF‐α ‐stimulated RA‐FLS

**Figure 3:**
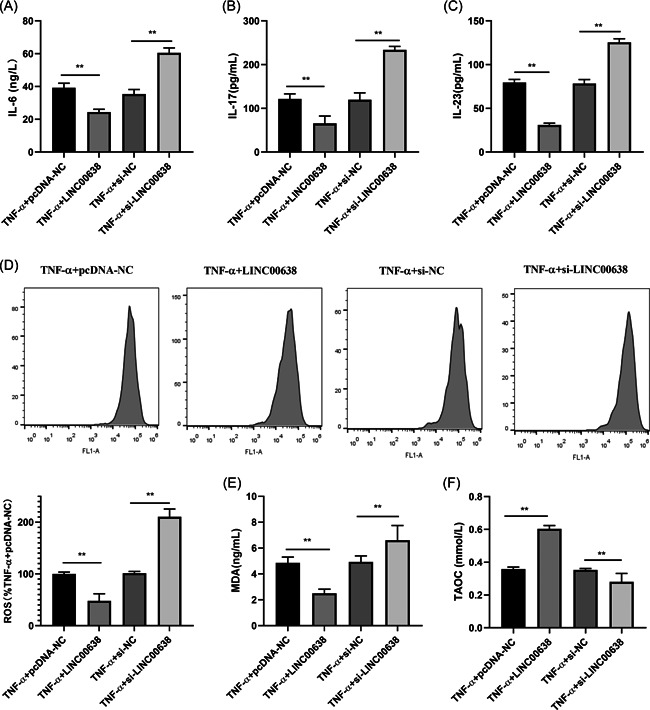
Effects of LINC00638 on cytokines and oxidative stress of TNF‐α‐stimulated RA‐FLS. (A–C) Expression of IL‐6, IL‐17, and IL‐23 was measured by ELISA. (D) The expression level of ROS was detected by flow cytometry. (E, F) Expression of MDA and TAOC was determined by biochemical assay. IL‐6, interleukin‐6; IL‐17, interleukin‐17; IL‐23, interleukin‐23. *p < .05, **p < .01. ELISA, enzyme‐linked immunosorbent assay; IL, interleukin; MDA, malondialdehyde; ROS, reactive oxygen species; TAOC, total antioxidant capacity

ELISA results showed that overexpression of LINC00638 inhibited the expression of IL‐6, IL‐17, and IL‐23 in TNF‐α ‐stimulated RA‐FLS. However, silencing LINC00638 increased the expression of these inflammatory cytokines (Figure 3A–C). In addition, we measured ROS by flow cytometry and the levels of both MDA and TAOC by biochemical method. The results showed that the transfection of pcDNA‐LINC00638 significantly reduced the levels of ROS and MDA, while increasing the expression of TAOC. On the contrary, the silencing of LINC00638 led to opposite results (Figure 3D–F).

### 3.6 LINC00638 can regulate Nrf2/HO‐1 pathway in TNF‐α‐stimulated RA‐FLS

**Figure 4:**
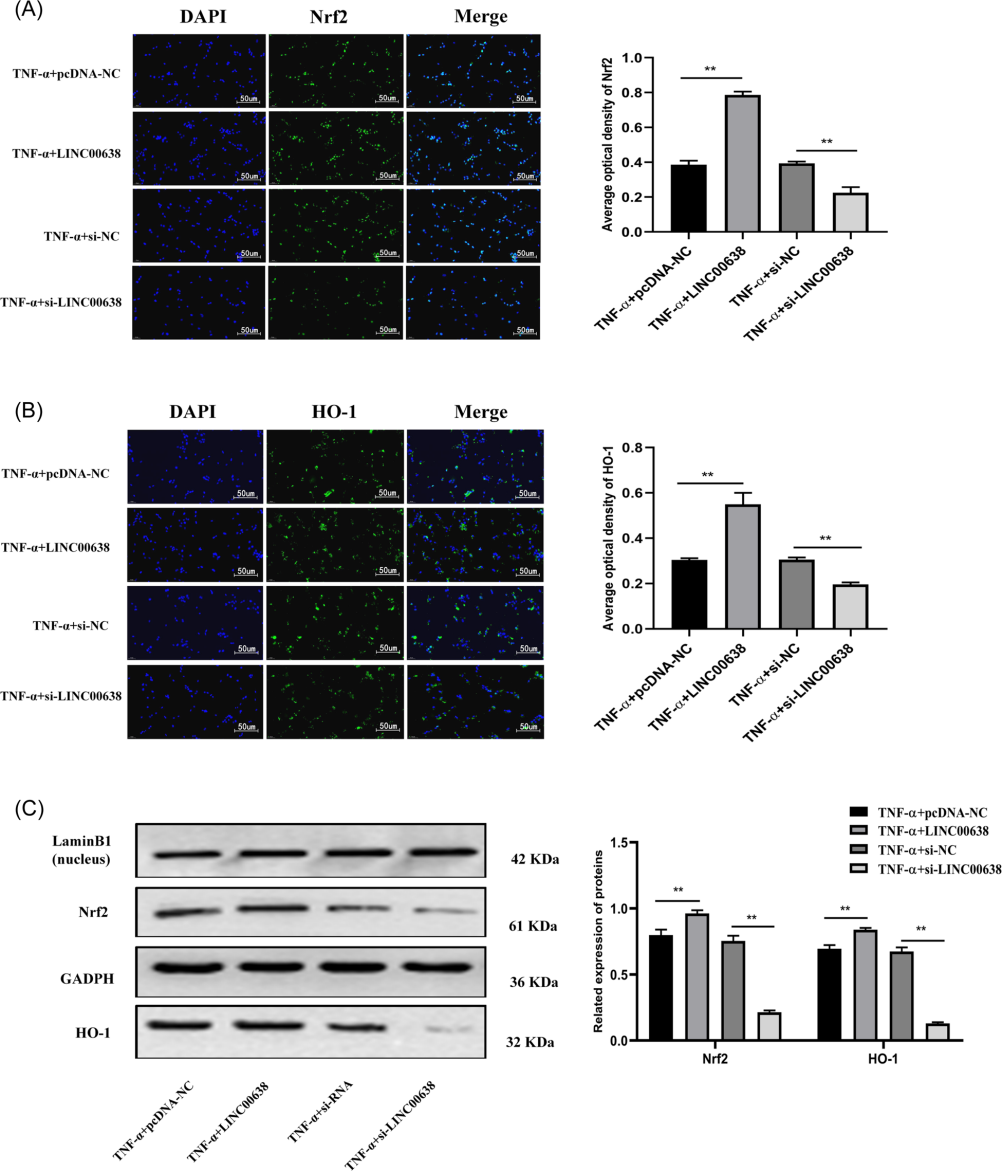
LINC00638 regulates Nrf2/HO‐1 pathway in RA‐FLS stimulated by TNF‐α. (A–B) Expression of Nrf2 and HO‐1 proteins was measured by Immunofluorescence (scale bar, 50 μm). (C) Expression of Nrf2 and HO‐1 proteins was measured by western blot. *p < .05, **p < .01. HO‐1, heme oxygenase 1; Nrf2, nuclear factor erythrocyte derived 2 related factor 2; RA, rheumatoid arthritis; TNF, tumor necrosis factor

To verify whether LINC00638 can regulate the activation of Nrf2/HO‐1 pathway, IF and western blot analysis was used. The results of IF assay showed that transfection of pcDNA‐LINC00638 can promote the expression of Nrf2 and HO‐1, while transfection of si‐LINC0068 reduced Nrf2 and HO‐1 levels (Figure 4A,B). Western blot analysis indicated that overexpression of LINC00638 promoted Nrf2 and HO‐1 levels, while silencing of LINC00638 exhibited an opposite effects (Figure 4C).

## 4 DISCUSSION

With the development of sequencing technology, lncRNAs have received significant attention. For example, lncRNAs can be used as novel markers of diseases and the regulatory networks of specific lncRNAs have been studied previously.^18^, ^19^ A number of recent reports have shown that several dysregulated lncRNAs are involved in the inflammatory response, cellular growth and apoptosis of RA.^20^, ^21^ However, there are few reports on the regulation of oxidative stress by lncRNAs in RA and further studies related to their regulation mechanisms are needed. Based on in vivo results, it was found that LINC00638 was expressed at lower levels in PBMC of RA patients. Moreover, the levels of LINC00638 in PBMCs of RA patients were negatively correlated with the levels of inflammatory factors and oxides in serum. Therefore, to explore whether LINC00638 can also participate in the inflammatory response and oxidative stress in RA, we conducted a detailed analysis in RA‐FLS. In vitro, LINC00638 was expressed at lower levels in RA‐FLS, which was consistent with the level of PBMC in RA patients. In addition, overexpression of LINC00638 inhibited the proliferation and migration of TNF‐α‐stimulated RA‐FLS, activate the Nrf2/HO‐1 pathway, decreased the levels of IL‐6, IL‐17, IL‐23, ROS as well as MDA, and increased the level of TAOC. In contrast, silencing of LINC00638 exhibited opposite effects.

A previous study has shown that the expression of LINC00638 was related to the disease activities of the RA patient, but no specific regulatory mechanisms were identified.^22^ The data from our experiments revealed that LINC00638 expression was significantly decreased in RA‐FLS and PBMC, which clearly suggested that LINC00638 can play a major role in the development of RA. Oxidative stress is known to be associated with inflammation, and both factors have been found to be strongly linked with the onset of RA.^23^ Free radicals such as ROS and reactive nitrogen are considered as effective mediators of inflammation,^24^ which can induce and aggravate the inflammatory response, while antioxidants play a crucial role in reducing inflammation. The findings of our study showed that the level of IL‐6, IL‐17, IL‐23, ROS, and HO‐1 was significantly increased, while that of TAOC was decreased in RA patients, thus indicating that there was an imbalance of immune‐inflammatory response and oxidative homeostasis. HO‐1 functions as a protective antioxidant enzyme in the human body. When the body is stimulated by oxidation, HO‐1 levels can be increased under stress, and it thus can play an important anti‐inflammatory and antioxidant role.^25^, ^26^ In addition, Spearman correlation analysis showed that the levels of LINC00638 were negatively correlated with DAS28, ESR, IL‐17, and ROS, thus suggesting that LINC00638 may be involved in RA immune‐inflammatory response and oxidative stress. Therefore, it is necessary to further explore the regulatory mechanisms of LINC00638.

FLS hyperplasia can promote RA inflammation and joint destruction.^27^ RA‐FLS has the ability to display tumor‐like cell invasion as well migration, can actively proliferate and produce different inflammatory cytokines.^28^ For instance, one study showed that LncRNA FOXD2‐AS1 can adsorb miR‐331‐3p as a sponge to promote the proliferation of FLS in RA.^29^ Moreover, another study revealed that Neohesperidin can reduce inflammation, oxidative stress and inhibit the proliferation of RA‐FLS.^30^ In this study, overexpression of LINC00638 can significantly inhibit the proliferation and migration of TNF‐α‐stimulated RA‐FLS while silencing of LINC00638 promoted proliferation and migration. Therefore, we concluded that LINC00638 can regulate both the proliferation and migration of RA‐FLS.

LncRNA can also regulate the inflammation and oxidative stress process of the various diseases in a variety of ways. For example, Gao et al.^31^ showed that lncRNA NKILA can reduce inflammation and oxidative stress via inhibiting the activation of nuclear factor kappa B pathway. In another study, Lv et al.^32^ also reported that lncRNA XIST can promote the formation as well as the development of kidney stones by mediating inflammatory response and oxidative stress with NLRP3/caspase‐1/IL‐1 signal pathway. This study showed that overexpression of LINC00638 could inhibit the expressions of IL‐6, IL‐17, IL‐23, ROS, MDA and increase the level of TAOC in TNF‐α‐stimulated RA‐FLS, while silencing LINC00638 displayed opposite effects. Therefore, LINC00638 can inhibit the level of inflammatory response and oxidative stress in RA‐FLS, and thereby improve the antioxidant capacity.

Nrf2 signaling pathway plays a vital role to regulate the cell redox homeostasis.^33^ Nrf2 is a major transcription factor regulating HO‐1, NQO1, and other antioxidant enzymes.^34^, ^35^ In this study, the data of IF and Western blot showed that transfection of pcDNA‐LINC00638 increased Nrf2 and HO‐1 expression in RA‐FLS, while transfection of si‐LINC00638 substantially decreased the levels of these proteins. This observation suggested that LINC00638 could regulate inflammation and oxidative stress in RA‐FLS by activating the Nrf2/HO‐1 pathway. Similarly, several other studies have elucidated potential correlation and mechanism of action of lncRNAs in the Nrf2/HO‐1 pathway of different diseases. For example, Zhang et al.^36^ showed that Pinoresinol Diglucoside could exert antioxidant and anti‐inflammatory effects through modulating Nrf2/HO‐1 pathway, thus alleviating brain injury in mice. In addition, Lv et al.^37^ reported that downregulation of lncRNA GAS5 can promote Nrf2/HO‐1 pathway and inhibit pyroptosis of human bronchial epithelial cells.

This study is associated with certain limitations. This study lacks in vivo experimental evidence that LINC00638 can play a specific role in regulating inflammation and oxidative stress in RA, which is also the focus of our future research, such as conducting animal experiments related to RA. In addition, PBMCs is a cell population composed primarily of the lymphocytes, monocytes and dendritic cells. Among them, different subtypes of cells can cause heterogeneity in the population. In this study, the expression of LINC00638 was found to be decreased in PBMC of RA patients, but greater sample size and involvement of more study centers are required to verify this result.

## 5 CONCLUSION

In conclusion, our findings provide a potential molecular understanding of the pathogenesis of inflammation and oxidative stress associated with RA. We observed that LINC00638 was expressed at low levels in RA patients and correlated with inflammation and oxidative stress. In addition, overexpression of LINC00638 can effectively reduce the proliferation and migration of RA‐FLS, and activate the Nrf2/HO‐1 pathway to inhibit the inflammation as well as oxidative stress in RA.

## AUTHOR CONTRIBUTIONS

**Yanqiu Sun, Jian Liu, and Jianting Wen**: were involved in the experimental design. **Xianheng Zhang, Xiang Ding, and Xiaolu Chen**: participated in the collection and data analysis of clinical specimens. **Yanqiu Sun, Dan Huang, and Qin Zhou**: conducted the research. **Yanqiu Sun**: performed manuscript writing. All authors reviewed the contents of the final manuscript.

## CONFLICTS OF INTEREST

The authors declare no conflicts of interest.

## ETHICS STATEMENT

This study was carried out after approval by the Ethics Committee of the First Affiliated Hospital of Anhui University of Traditional Chinese Medicine (Hefei, China) (2019AH‐12). The processing of clinical samples strictly complied with the Declaration of Helsinki, and all subjects signed the informed consent.

## Data Availability

The data used in this study are available from the corresponding author at reasonable request.
